# Drug fever—an immune-mediated delayed type hypersensitivity reaction to Vinca alkaloids in pediatric oncology patients, possibly mediated by cysteinyl leukotrienes

**DOI:** 10.3389/falgy.2024.1361403

**Published:** 2024-08-06

**Authors:** Mona I. Kidon, Soad Haj Yahia, Gadi Abebe-Campino, Nancy Agmon-Levin, Michal Yelon

**Affiliations:** ^1^ Pediatric Allergy Unit, Edmond and Lilly Safra Children's Hospital Safra, Sheba Medical Center, Ramat Gan, Israel; ^2^ Allergy and Clinical Immunology Unit, Sheba Medical Center, Ramat Gan, Israel; ^3^ Faculty of Medicine, Tel Aviv University, Tel Aviv, Israel; ^4^ Pediatric Hemato-Oncology, Edmond and Lilly Safra Children's Hospital, Sheba Medical Center, Ramat Gan, Israel

**Keywords:** fever, adverse drug reactions, chemotherapy, leukotrienes, oncology

## Abstract

**Background:**

Drug hypersensitivity reactions are common in pediatric hemato-oncology patients due to multiple factors including immune compromise and pharmacological complexities. Fever can signify severe delayed-type hypersensitivity reactions such as drug reaction with eosinophilia and systemic symptoms (DRESS) or drug-induced hypersensitivity syndrome (DIHS). The etiology of fever as an isolated hypersensitivity reaction to chemotherapeutic agents not fully understood. Here, we report three children with intracranial neoplasms experiencing recurrent febrile reactions following Vinca alkaloid-based chemotherapy, mitigated by cysteinyl leukotriene receptor antagonist therapy.

**Methods:**

We present a series of pediatric patients with diverse intracranial neoplasms who developed recurrent fever episodes after multiple courses of Vinca alkaloid-based chemotherapy. Treatment involved prophylactic and post-chemotherapy administration of a cysteinyl leukotriene receptor antagonist to prevent fever episodes and enable completion of chemotherapy regimens without protocol modifications or desensitization.

**Results:**

All three patients experienced fever consistent with delayed-type hypersensitivity reactions to Vinca alkaloids. Prophylactic use of the leukotriene antagonist Montelukast successfully prevented fever recurrence, allowing uninterrupted completion of chemotherapy courses.

**Conclusion:**

Our findings suggest that Montelukast, a leukotriene antagonist, may be beneficial in managing fever as a delayed-type hypersensitivity reaction to Vinca alkaloids in pediatric patients. Further research is warranted to elucidate the underlying mechanisms and leukotriene pathways involved in drug-induced fever reactions.

## 1 Introduction

Allergic hypersensitivity reactions to drugs in children in general are infrequent, and although the prevalence of self-reported drug allergies in the pediatric age group ranges from 2.9% to 16.8% (1), only about 4% of these suspected drug allergies are confirmed after appropriate diagnostic work-up. By contrast, the prevalence of drug hypersensitivity reactions in pediatric oncology patients is significantly increased, estimated between 14% and 68%, with almost a quarter diagnosed as severe (2).

Fever is a frequent manifestation of severe delayed-type hypersensitivity reactions such as drug reaction with eosinophilia and systemic symptoms/drug-induced hypersensitivity syndrome (DIHS/DRESS). However, the estimated prevalence of fever as an isolated hypersensitivity reaction is only 5%–10% (3), while the actual reporting is hampered by frequent misdiagnosis and mislabeling, so that only in 0.05% of adverse drug reactions (ADRs) reported to the French National Pharmacovigilance Database fever was an isolated phenomenon (4).

By contrast, drug fever is a known complication of anti-cancer medications, potentially causing quite severe hyperthermia usually on day number 3–4 after a course of chemotherapy (5). In adults, the risk of developing drug fever after cancer medication is increased in females and in younger patients, increases with the number of drugs prescribed, can occur within 48 h to several weeks after drug initiation and usually resolve several days after drug cessation and recur after re-exposure in the same individual (6).

These features of fever following anti-cancer therapies identify drug fever, in most cases, as an immune-mediated adverse reaction. Since it is indistinguishable from a potentially severe bacterial infection in a patient with a compromised immune system, it requires initial hospitalization with systemic antibiotics and monitoring, with subsequent impairment of quality of life, further disruption of the normal daily routine, unnecessary medications, and costs (4).

When presenting as part of the complex of DIHS/DRESS, the pathogenesis of fever most likely involves delayed-type cellular responses, in particular CD4+ and CD8+ lymphocytes and Thymus and activation regulated chemokine (TARC) producing dendritic cells, recruiting CCR4+ Th2T-cells to the skin, in turn responsible for the production of IL-5. This induces differentiation, activation, and migration of eosinophils to the peripheral blood and tissues. Tumor necrosis factor-α and interferon-γ are also known to be elevated in acute DIHS/DRESS (7).

By contrast, the mechanisms involved in the pathogenesis of drug fever as an isolated phenomenon in general, as well as those involved specifically after exposure to chemotherapeutic agents, are not clearly defined. Possible mechanisms of action in chemotherapy-related drug fever could be attributed to one or a combination of five broad categories, as follows: (1) altered thermoregulatory mechanisms; (2) drug administration-related fever and/or tremors, with accelerated cell death and an increased burden of cellular debris, heat shock proteins, and cytokines; (3) fevers relating to the pharmacologic action of a drug; (4) idiosyncratic reactions; and (5) possibly a common mechanism for the development of fever in this setting, i.e., an immune-mediated hypersensitivity reaction, in which case the likelihood of a repeat reaction after a repeated exposure would be expected to be quite high.

## 2 Methods

We describe a series of children with recurrent bouts of unexplained fever after sequential courses of Vinca alkaloid-based chemotherapy, treated with pre- and post-medication with a leukotriene (LTE) receptor antagonist, enabling the completion of their prescribed courses without further episodes and without the need for protocol modification or desensitization.

The possible mechanism is discussed as well as the need for further research.

## 3 Results

### 3.1 Patients

#### 3.1.1 Patient 1

A 10-year-old girl was diagnosed with NF-1 and an intracranial chiasmatic glioma. She began treatment with vincristine and carboplatin in 2016, but the tumor progressed. In February 2017, her treatment was changed to vinblastine. In June 2017, she experienced a mild fever (up to 38.7°C) and neutropenia (800 cells/ml) within 24 h after the 15th full dose of vinblastine. She showed no specific localized symptoms but appeared unwell, leading to hospitalization. Blood and urine cultures were taken, and she received IV piperacillin for 3 days until cultures were negative.

Similar fever episodes occurred after the 16th, 17th, and 18th weekly doses of vinblastine, occurring 24–36 h post-infusion. The patient was started on montelukast 20 mg on the day of vinblastine infusion and for the following 48 h. This treatment completely eliminated fever and chills, allowing her to complete the full course of 72 weekly treatments without further complications.

#### 3.1.2 Patient 2

A 7-year-old boy diagnosed with NF-1 and optic glioma began treatment in 2019 with vincristine and carboplatin. During the 8th infusion, he experienced an immediate reaction characterized by a sore throat and urticaria, which were managed symptomatically. Subsequently, the treatment was switched to Vinblastine, and he received seven weekly courses with adjusted dosages. After the 8th course of vinblastine, the patient developed fever and chills, leading to hospitalization. Blood and urine cultures were obtained, and he received IV piperacillin for 3 days until cultures returned negative. Similar fever episodes occurred 24–36 h after the 9th and 10th weekly doses of vinblastine. To manage these reactions, the patient was initiated on montelukast 20 mg concurrently with vinblastine infusions and for the following 48 h. Following this adjustment, he continued his vinblastine treatment without further complications.

#### 3.1.3 Patient 3

A 10-year-old boy with a background of chronic allergic rhinitis and asthma was diagnosed with intracranial juvenile pilocytic astrocytoma (JPA) with BRAF fusion. The treatment began with vinblastine in July 2017. In May 2018, following four episodes of fever, each occurring 24 h post-vinblastine infusion and with negative cultures, the patient commenced prophylactic montelukast at a daily dose of 5 mg. Subsequently, there were no further febrile episodes, allowing the successful completion of the entire course of vinblastine treatment.

## 4 Discussion

LTEs are a family of products of the 5-lipoxygenase pathway of arachidonic acid (AA) metabolism. Their production during health is usually in balance with various prostaglandins (PGs), produced from AA through the action of cyclooxygenases. Formerly called slow reactive substance of anaphylaxis, the cysteinyl leukotrienes C4, D4, and E4 are involved in inflammatory processes in asthma and other type 2-related diseases, are synthesized in leukocytes and mast cells, and modulate leukocyte chemotaxis, smooth muscle contraction, and immediate inflammatory responses (8).

The cysteinyl leukotrienes are conjugates of the eicosanoid fatty acid epoxide, leukotriene A4 (LTA4), with glutathione or glutathione cleavage products. The primary cysteinyl leukotriene product, leukotriene C4 (LTC4), is secreted by activated mast cells and causes a rapid wheal and flare reaction in the skin of the type associated with insect bites. After secretion, LTC4 is converted to LTD4 and LTE4 by sequential peptidase digestion of the conjugated glutathione moiety. These compounds show generally similar activities to LTC4, but have different receptor selectivity (9).

Vinca alkaloids are a class of naturally occurring compounds found in the periwinkle plant (*Catharanthus roseus*). They are used in chemotherapy to treat a variety of cancers, including leukemia, lymphoma, and solid tumors. Their mechanism of action is by inhibiting the formation of microtubules, which are essential for cell division. Vinca alkaloids are very effective at killing cancer cells, but they can also cause serious side effects, such as hair loss, nerve damage, and bone marrow suppression. These side effects are usually temporary, but they can be severe in some people.

Eicosanoid mediators of inflammation and platelet function are released as a result of tissue damage, secondary to the action of Vinca alkaloids to prevent the elongation of microtubules in dividing cells. Thromboxane A2 is a product of the cyclooxygenase pathway secreted by platelets that modulates vascular tone and is a potent secondary agonist of platelet activation (10).

It has been previously shown that the leukotriene pathway can influence the effect of vincristine on Multi Drug Resistance (MDR) cells (11), and therefore the idea that anti-leukotriene medications such as montelukast would be able to modify adverse reactions to this class of drugs.

We are reporting on a group of children with intracranial tumors, with recurrent febrile episodes after repeated courses of Vinca alkaloids, treated with a leukotriene receptor antagonist, enabling the eradication of the febrile reaction and the completion of the required course of chemotherapy.

Variations in montelukast dosage were notably influenced by the presence of asthma diagnoses. Patients 1 and 2, who did not have a history of asthma, did not routinely receive montelukast. Therefore, they were administered a higher dose of montelukast concomitant with and after vincristine therapy, similarly to pre/post-treatment protocols during drug rechallenge/desensitization. By contrast, patient 3, diagnosed with asthma, received the standard age-appropriate dose as a continuous/permanent treatment.

As with individual reactions to the therapeutic effects of medications, individual adverse drug reactions most likely are mainly driven by genetic factors and may be unraveled in the future by the “new science” of pharmacogenomics (12).

In these patients, hyperthermia appears to be integral to a delayed hypersensitivity reaction to Vinca alkaloids. Hence, pro-inflammatory cytokines implicated in such reactions are suspected contributors to the underlying pathophysiological mechanism. Previous studies have shown that leukotrienes participate in immediate-type hypersensitivity reactions, prompting the recommendation of anti-leukotrienes during drug desensitization protocols (13).

The successful resolution of severe drug fever induced by Vinca alkaloids through the use of leukotriene receptor antagonists in our patients underscores the immune-mediated nature of this reaction. The use of leukotriene receptor antagonists not only enables safe and timely intervention, ensuring adherence to chemotherapy protocols, but also prompts further investigation into the mechanisms of drug fever. This approach highlights the potential implications of leukotriene pathways in both delayed-type hypersensitivity reactions and immediate adverse drug reactions (ADRs).

Understanding individual hypersensitivity responses to specific drug classes remains a significant unmet need in both pediatric and adult populations, necessitating further research.

## Data Availability

The raw data supporting the conclusions of this article will be made available by the authors, without undue reservation.
